# A Dynamic Analysis of Smart and Nanomaterials for New Approaches to Structural Control and Health Monitoring

**DOI:** 10.3390/ma16093567

**Published:** 2023-05-06

**Authors:** Wael A. Altabey, Mohammad Noori

**Affiliations:** 1 International Institute for Urban Systems Engineering (IIUSE), Southeast University, Nanjing 210096, China; 2 Department of Mechanical Engineering, Faculty of Engineering, Alexandria University, Alexandria 21544, Egypt; 3 Department of Mechanical Engineering, California Polytechnic State University, San Luis Obispo, CA 93405, USA; 4 School of Civil Engineering, University of Leeds, Leeds LS2 9JT, UK

## 1. Introduction and Scope

During recent years, remarkable progress has been made in the development of new materials. Advanced structured materials, including smart and nano materials, have opened up new engineering possibilities because of their specific properties (chemical, mechanical, and physical) that are not found in nature, and can be significantly changed by a user in a controlled manner to make them appropriate for certain applications. Due to their unique properties, smart and nano materials have been of interest in countless areas of technical application, in various systems and structures, including intelligent and adaptive sensing or actuation, as well as active control. An understanding of the relationships between their structures and properties is of crucial importance for the practical utilization of these materials.

**Figure 1:**
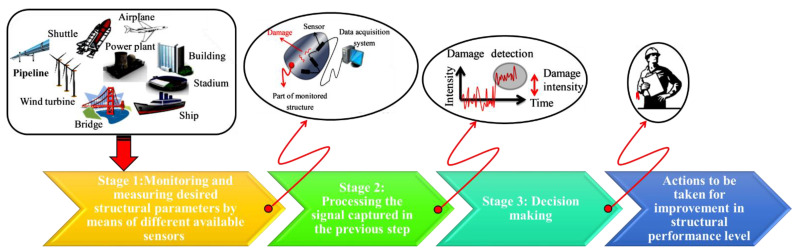
Integrating structural control and health monitoring.

Over the past several years, a series of approaches for progress in structural control and healthy monitoring have left paramount impacts on our everyday lives. This has shaped the framework of many engineering fields. Given the current state of quantitative and principled methodologies, nowadays, it is possible to rapidly and consistently evaluate the structural safety of mechanical systems, industrial machines, and modern concrete buildings, etc., to test their capability for serving their intended purpose. However, unsolved, problematic, and new challenges exist. Unmolded nonlinearities, ineffective sensor placement, and the effects of confounding influences due to operational and environmental variabilities still harm the effectiveness of the state of structural control and healthy monitoring systems. A typical integrating structural control and health monitoring system is shown in Figure 1.

The aim of this Special Issue is to gain new, unique knowledge about the relationships between the structures and physico-mechanical and chemical properties of new materials, including finding ways to structure the control and development of new methods for structural healthy monitoring. Another goal is to gather the main contributions of academics and practitioners in mechanical, aerospace, and civil engineering to provide a common ground for improvements to these approaches to structural control and healthy monitoring, by using the unique properties of smart and nano materials. Studies concerning sensor technologies, vibration-based techniques, artificial-intelligence-based methods, and related fields are all welcome, in both numerical and experimental form.

The keywords of this Special Issue are:

- Structural health monitoring;
- Structural control;
- Smart materials and structures;
- Nanomaterials and nanocomposites;
- Sensors and actuators;
- Energy harvesting;
- Artificial intelligence;
- Damage detection;
- System identification;
- Machine learning;
- Sensor placement;
- Intelligent structure systems.

Evidently, the articles accepted for publication will cover all the topics and it is expected that the manuscripts published here are of interest to researchers working in improving the materials utilized in structural control and health monitoring.

## 2. Contributions

The manuscripts of this Special Issue can be classified according the topics provided in Table 1, based on some reviews of the literature.

## 3. Conclusions and Outlook

Considering the knowledge of the guest editors of this Special Issue, the preservation of the structural integrity of the damaged parts is distinguished by a control and repair procedure through establishing the smart and nanomaterials effect, in which local moment and force are induced in these materials by applying the converse effect of the structure, in order to break the increase in stress and strain levels due to the external load, thus decreasing the criticality of the damage. Additionally, entering structural health monitoring (SHM) and artificial intelligence (AI) techniques into the procedure of controlling and repairing the damage to structures will definitely highly affect the avoidance of a premature collapse of mechanical and civil structures such as pipelines, houses, bridges, aerospace, and offshore platforms. In this Special Issue, we underwent efforts to collect contributions from active researchers in the fields of structural control and health monitoring, and also mechanical, structural, electrical, material, and other engineering fields. It will act as a platform for sharing. Furthermore, researchers may provide transparent views and indices for their research areas through challenges and opportunities. In short, this sharing can help researchers to develop new ideas, particularly in the early stages of this research field.

## Figures and Tables

**Table 1:** Information about topics within the Special Issue’s manuscript contributions.

Topic	Highlights	Refs.
Structural health monitoring techniques	SHM is a new field of research and development that emanated from smart materials and structures. SHM has attracted considerable attention in recent years for assessments in infrastructure and aerospace vehicle applications. The goal of SHM to develop automated systems can enable continuous monitoring, inspection, and the detection of damage to structures, to minimize the need for human labor.	[1,2]
Advances in structural control	The control of structures is a study in which smart and nanomaterials are utilized for various purposes. Due to their versatile applications, smart materials such as PZT transducers can control metallic and non-metallic structural components as the main object for controlling a structural component’s vibration, noise, and activity.	[3,4]
Smart materials and structures	A smart structure is a system containing multifunctional parts that can perform sensing, control, and actuation; it is a primitive analogue of a biological body. Smart materials are used to construct these smart structures, which can perform both sensing and actuation functions.	[5,6]
Advanced nanomaterials applications	Metallic nanoparticles are used as reinforcements in alloys for light constructions that have an appropriate resistance and hardness, mainly in the aerospace and automotive sector; for example, titanium nanoparticles are used as a steel alloy element and the resulting alloy shows improved properties with respect to resistance, ductility, temperature, and corrosion resistance.	[7]
Nanocomposite applications	A nanocomposite is a matrix to which nanoparticles have been added to improve a particular property of the material. The properties of nanocomposites have caused researchers and companies to consider using this material in several fields such as making flexible batteries, lightweight sensors, composites with even higher strength-to-weight ratios, speeding up the healing process for broken bones, impellers, and blades.	[8]
Nano pipes and films	Nano pipes and film systems are widely used in micro/nano-electro-mechanical sensors (NEMS/MEMS); they are among the most critical components in these sensors, such as pressure sensors that are exposed to stress and harsh environmental conditions.	[9]
Artificial intelligence application	In early applications, the algorithms of AI-based schemes were used for SHM and damage detections such machine learning, deep learning, and artificial neural networks.	[10,11,12]
Self-repair and self-assembly application	Self-repair and self-assembly materials have attracted attention due to their ability to regain their structure and function after damage. In recent years, significant progress has been made in achieving various functions through supramolecular chemistry. Self-repair materials are artificially created substances that have a built-in ability to automatically repair damage without any external diagnosis of the problem or human intervention.	[13]
Reconstruction techniques	Introducing smart materials as integral parts of civil structures or active or sensitive constructive elements opens up a wide range of abilities. In the context of building construction, several smart material methodologies are already available and commonly used, for example, piezoelectric transducers for SHM.	[14]
Energy harvesting	Piezoelectric energy harvesting is commonly applied in SHM due to its great ability to provide self-powered electronic wearable devices and wireless sensor networks. Piezoelectric converts mechanical energy into electricity with a high efficiency and ease of operation. Harvested power can be used in many medical and industrial applications such as pacemakers, bridge and building monitoring, and tire pressure monitoring techniques.	[15,16,17,18,19]
